# Employing Energy and Statistical Features for Automatic Diagnosis of Voice Disorders

**DOI:** 10.3390/diagnostics12112758

**Published:** 2022-11-11

**Authors:** Avinash Shrivas, Shrinivas Deshpande, Girish Gidaye, Jagannath Nirmal, Kadria Ezzine, Mondher Frikha, Kamalakar Desai, Sachin Shinde, Ankit D. Oza, Dumitru Doru Burduhos-Nergis, Diana Petronela Burduhos-Nergis

**Affiliations:** 1 Department of Computer Science & Technology, Degree College of Physical Education, Sant Gadge Baba Amravati University, Amravati 444605, India; 2 Department of Electronics and Computer Science, Vidyalankar Institute of Technology, Mumbai University, Mumbai 400037, India; 3 Department of Electronics Engineering, Somaiya Vidyavihar University, Mumbai 400077, India; 4 ATISP, ENET’COM, Sfax University, Sfax 3000, Tunisia; 5 Department of Electronics and Telecommunication Engineering, Bharati Vidyapeeth’s College of Engineering, Shivaji University, Kolhapur 416013, India; 6 Department of Mechanical Engineering, Datta Meghe College of Engineering, Mumbai University, Airoli, Navi Mumbai 400708, India; 7 Department of Computer Sciences and Engineering, Institute of Advanced Research, The University for Innovation, Gandhianagar 382426, India; 8 Faculty of Materials Science and Engineering, Gheorghe Asachi Technical University of Iasi, 700050 Iasi, Romania

**Keywords:** statistical features, inverse filtering, glottal flow, feature selection, voice disorder, wavelet transform

## Abstract

The presence of laryngeal disease affects vocal fold(s) dynamics and thus causes changes in pitch, loudness, and other characteristics of the human voice. Many frameworks based on the acoustic analysis of speech signals have been created in recent years; however, they are evaluated on just one or two corpora and are not independent to voice illnesses and human bias. In this article, a unified wavelet-based paradigm for evaluating voice diseases is presented. This approach is independent of voice diseases, human bias, or dialect. The vocal folds’ dynamics are impacted by the voice disorder, and this further modifies the sound source. Therefore, inverse filtering is used to capture the modified voice source. Furthermore, the fundamental frequency independent statistical and energy metrics are derived from each spectral sub-band to characterize the retrieved voice source. Speech recordings of the sustained vowel /a/ were collected from four different datasets in German, Spanish, English, and Arabic to run the several intra and inter-dataset experiments. The classifiers’ achieved performance indicators show that energy and statistical features uncover vital information on a variety of clinical voices, and therefore the suggested approach can be used as a complementary means for the automatic medical assessment of voice diseases.

## 1. Introduction

A key component of human conversation is speech signals. However, the multiple laryngeal illnesses harm the parts of the voice box that are responsible for speech, creating an abnormal voice that is unfit for everyday uses [1]. As a result, it is essential to identify the source of a voice anomaly as soon as possible. Traditional methods for examining voice dysfunction in medical practice are subjective and invasive. The grade, roughness, breathiness, asthenia, and strain (GRBAS) and the Consensus Auditory Perceptual Evaluation–Voice (CAPE-V) scales are used in the traditional perceptual analysis [2]. Such perceptual examination is subjective, imprecise, and necessitates expert professionals. Laryngoscopy, stroboscopy, and endoscopy are methods used in the obstructive clinical process to observe the larynx’s inner structure [3]. These testing methods necessitate a skilled ear, nose, and throat doctor and a cutting-edge laboratory outfitted with instruments. The patient may perform (present) a reflex response in the orinasal cavity while using these methods, resulting in erroneous assessments [4]. For the early and reliable assessment of laryngeal illnesses, an effective alternative approach focused on an acoustical analysis of the clinical voice may be possible. There are two main types of established voice pathology identification frameworks: classical pipeline frameworks and newer end-to-end frameworks [5]. The conventional pipeline scheme is made up of two parts: extraction of features and identification [5,6]. The feature extraction section attempts to obtain useful information from speech records. The attribute sets employed by the research community for the identification of voice abnormalities can be divided into four types: (1) perturbation attributes; (2) spectral and cepstral attributes; (3) complexity attributes; and (4) glottal attributes [5,6,7]. A classification algorithm is used in the detection phase to categorize the source speech as normal or pathological. The numerous acoustic analysis-based approaches focused on machine learning are addressed in [8]. In the end-to-end voice analysis approach, huge speech records are required. However, because of a scarcity of records, end-to-end schemes have still not been extensively used in the research area of voice pathology identification [6,7]. The analysis of pathological voice is challenged by the scarcity of available speech samples, as these samples come from patients whose illness may be so severe that only short recordings are possible. As a result of the scarcity of the large quantity of training samples required to educate end-to-end schemes, the conventional pipeline scheme remains a workable option [7].

Perturbation metrics detect the occurrence of amplitude and frequency instability in the voice signal induced by the abnormal vibration of vocal tract tissues and the inability of glottal closure. Shimmer and jitter are the two most common metrics of this category employed in auditory analysis; however, there are numerous variants of jitter and shimmer in use to recognize voice pathologies. The framework based on these features is presented in [9]. The recordings of sustained vowels as well as continuous speech mined from the Massachusetts Eye and Ear Infirmary (MEEI) dataset were employed to carry out the voice assessment task. Two sets of experiments were carried out: in the first set, an individual metric is chosen, while in the second set, a combination of all nine metrics is considered. The categorization was more correct when the individual metrics were selected from sustained verbal recordings than with those based on continuous speech samples. The accuracy rate increased to 96% by considering a combination of the metrics. The correlation dimension is used along with the two main perturbation metrics to separate laryngeal paralysis subjects from healthy ones [10]. The spectral jitter estimation method is used to obtain the jitter, since it is resilient against inaccuracies in estimating the pitch time. The voice recordings from the MEEI and Principe de Asturias (PdA) datasets are characterized. For the decision making, a separate threshold is decided for sustained vowel and running speech-based jitter. The area under the curve (AUC) obtained 0.94 and 0.84 for the jitter extracted from vowels for MEEI and PdA, respectively. However, the achieved AUC for running speech is 0.96 [11]. Other widely accepted disturbance metrics that assess the existence of aspiration noise include the harmonic-to-noise ratio (HNR), normalized noise entropy (NNE), voice turbulence index, and glottal-to-noise excitation (GNE) ratio. To assess the noise elements embedded in the pathological audio signal, an NNE metric is introduced and its efficacy in the diagnosis of larynx diseases is examined with 250 vocal samples (64 normal people and 186 sufferers with diverse laryngeal illnesses) [12]. To quantify such short-term perturbation metrics, precise fundamental frequency (Fo) mining is required. The precise estimation of the Fo in pathological voice is difficult [13,14], so all of these metrics are rejected from the attributes endorsed by the American Speech–Language–Hearing Association [15].

Spectrum and cepstrum-derived parameters have been widely adopted for voice disorder assessment, as these measures are usually simple to estimate and do not require an F0 calculation [14,16]. These measures have the potential to characterize both continuous speech and steady vowels. The widely accepted attributes in this type are mel-frequency cepstral coefficients (MFCC), linear predictive coding coefficients (LPC), linear predictive cepstral coefficients (LPCC), perceptual linear prediction (PLP), and cepstral peak prominence (CPP). In [17], auditory processed spectrum (APS) and cepstral coefficients based on all-pole model (APCC) measures are used for the detection and labeling of voice pathologies. The running speech recordings mined from the MEEI dataset were used to test the proposed method. The best-achieved voice pathology detection rate is 98.22% and 99.56% and the classification rate obtained is 93.33% and 89.33% with APS and APCC measures, respectively, using the Gaussian mixture model (GMM).

MFCC and their first (Δ) and second (ΔΔ) derivatives, along with the energy, are used as features for voice assessment in [18]. The proposed system is assessed using recordings of the sustained vowel /a/ quarried from the MEEI dataset. For decision making, multilayer perceptron (MLP) and learning vector quantization (LVQ) detectors are used. The best detection rate attained is 96% using LVQ detector and has performed better than MLP. The optimization of the preceding algorithm is addressed by Godino-Llorente et al. [19], which replaced the previous classification technique with a GMM and uses the extended subset speech recording from MEEI. In addition, to analyze the impact of derivatives in the voice pathology detection process, a feature selection approach centered on the F-Ratio and Fisher’s discriminant ratio is used. The authors noted a detection efficiency of 94%, suggesting that the addition of the ΔΔ derivative does not further boost the system’s accuracy. Then again, MFCC attributes as well as the same MEEI speaker audio files are selected in [20]. Numerous methodological problems are addressed with regard to developing systems that identify pathologies automatically. With the suggested technique, which is intended to enable comparisons with other relevant literature, an artificial neural network (ANN) efficiency of 90% is achieved. Ali et al. [21] have conducted various intra- and cross-dataset tests to verify the efficacy of the MFCC-based framework for voice impairment assessment. The system is tested using three diverse datasets: MEEI, SVD, and AVPD. The simulation results show that the MFCC measure-based system’s performance differs from dataset to dataset. The recognition accuracy for the intra-database varies considerably from 72% to 95% and for the inter-database varies from 47% to 82%. The results conclude that existing traditional speech attributes are not strongly linked with speech and are therefore not credible for screening and diagnostic tools. The authors of [14] tested the diagnostic importance of spectral/cepstral measurements, namely, cepstral peak prominence (CPP) and low–high spectral ratio (LHr) to distinguish pathological from non-pathological speech using sustained vowel and running speech records from the MEEI. The CPP (AUC = 0.95) performs better than LHr (AUC = 0.74) in diagnosis when these parameters are extracted from sustained vowels. A similar trend is observed when these parameters are extracted from running speech recordings, i.e., the AUC = 90 achieved with CPP and the AUC = 79 achieved with LHr.

Several researchers have found that certain nonlinear processes occur in speech production and cannot be characterized by traditional metrics such as shimmer, jitter, GNE, HNR, NNE, MFCC, LPCC, and PLP [22]. This is partly related to nonlinearities linked with pressure in the glottis, stress–strain curves of vocal cord muscle tissues, and vocal cord collision [23]. Complex metrics have been developed to characterize the nonlinearity in the audio signals. The correlation dimension (D2) is one of the most-studied complexity attributes of automatic voice quality evaluation systems. For example, the authors in [24] use it to evaluate signals as per their categorization in a dataset of 122 pathological records of the vowel /a/. The results show that D2 uncovers substantial differences between all kinds of signals. Subsequently, the authors of [11] considered D2 with jitter and shimmer to examine the prolonged vowels of persons with normophonic and unilateral laryngeal paralysis in an MEEI dataset subset (57 normal and 67 pathologic). Jitter and shimmer are only measured in nearly periodic sounds, resulting in considerable distinctions in both abnormal and normal voice groups (AUC = 0.7 for jitter and AUC = 0.8 for shimmer). D2 is determined for all voices having clear differences among pathological and normal states (AUC = 0.74). Studies suggest that perturbation and complexity metrics may categorize speech signals owing to their complementarity. A further investigation, including D2, jitter, shimmer, and second order entropies, is reported by Zhang and Jiang [25]. For the study of continuous speech production, the vowel /a/ and the vowels derived from the reading passage of the MEEI corpus are used. The findings revealed the ability of complexity attributes to distinguish among disordered and normal speech cases. On the other hand, perturbation metrics worked well with sustained vowels, but should be applied with precaution in vowels derived from continuous speech. Little et al. [26] presented a couple of new complexity attributes for the diagnosis of voice impairments: recurrence period density entropy (RPDE) and detrended fluctuations analysis (DFA). The recognition rate of 92% was attained with the proposed new attributes by employing a quadratic discriminant analysis for classification and bootstrap resampling for validation in the MEEI dataset. This recognition rate is greater than that attained by integrating other conventional parameters that do not surpass 81%. Two new entropy attributes based on the Markov models introduced in [27] are: Renyi HMM entropy and Shannon HMM entropy. These new attributes have been considered to characterize the MEEI dataset, together with nine other complexity attributes, three perturbation parameters, and MFCC. With all combined parameters, an accuracy of 98% is attained using a GMM.

Despite the fact that spectral and cepstral attributes have really been extensively employed in the assessment of voice impairment, it is important to note that they are quite likely to portray the vocal tract system than variations in the vibratory pattern of the vocal folds directly caused by voice illnesses. As a result, glottal source attributes derived from the glottal wave have a high research value. Forero et al. [28] captured the vocal tract excitation by glottal inverse filtering (GIF). Sixteen time instant-based, quotient-based, and frequency domain glottal metrics are extracted from the glottal wave and 12 MFCC extracted from speech signal to carry out the voice assessment. Three classes of subjects, namely, vocal fold paralysis, nodule, and healthy, are considered for the experiments. The classification accuracy attained with glottal parameters is 95.80%, 82.00%, and 96.20%; however, with the MFCC parameters, these values are 75.20%, 87.00%, and 80.00% using ANN, HMM, and SVM respectively. With the fusion of glottal and MFCC parameters, the achieved classification rate is 96.60%, 92.00%, and 97.20% using ANN, HMM, and SVM, respectively. Similarly, in [29], the authors proposed a voice disorder identification and classification system using an interlaced derivative pattern (IDP). The authors compared the IDP based system with MDVP and MFCC parameters. The suggested scheme attains average accuracies of 99.20%, 93.20%, and 91.50% in disorder identification tests using the SVM classifier with recordings from MEEI, SVD, and AVPD, respectively. In [30], different time-based, frequency-based, and Liljencrants–Fant (LF) model-based attributes were mined from the glottal pulse-form applying the well-known Aalto Aparat voice inverse filtering and parameterization tool. The normal pitch utterance of the sustained vowel /a/ mined from German, English, Arabic, and Spanish voice records is used. The highest rates of voice disorder identification achieved are 99.80%, 99.70%, 99.80%, and 99.80% respectively, over SVD, PdA, AVPD, and MEEI. The best voice illness categorization rates attained are 90.80%, 99.30%, 99.80%, and 90.10% for SVD, PdA, AVPD, and MEEI, respectively. The LF model-based attributes have demonstrated good discrimination power compared to other attributes for the assessment of voice disfunction.

This paper proposes an acoustical assessment-based technique for assessing voice illnesses induced by a malfunctioning vocal fold(s). A multi-resolution transform, such as a stationary wavelet transform (SWT), can detect the voice produced due to disorder since it has a significant amplitude fluctuation of extremely low scale. Sub-band distribution can identify unusual spectrum distribution, speech non-periodicity, and unexpected energy variations in the speech signal [31]. This is how we came up with the idea for using SWT in our research.

## 2. Materials and Methods

### 2.1. Voice Disorder Databases

This article employs four distinct language voice disease records. Table 1 shows the number of voice signals in each database, and Table 2 shows the database characteristics. The speech records with sustained vowel/a/are mined from these databases. Since the sampling frequency of each database varies, all speech records were resampled to 22.05 KHz to create a consistent (unique) sampling frequency across all datasets.

### 2.2. Proposed Speech Pathology Assessment Technique

**Figure 1:**
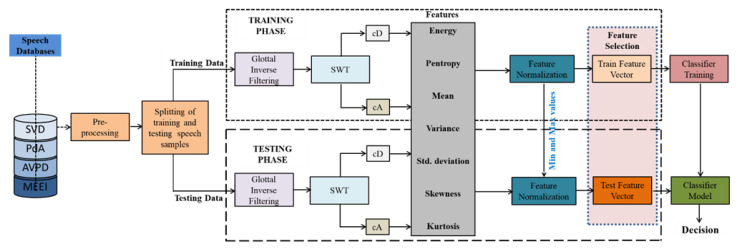
Voice disorder assessment system.

**Figure 2:**
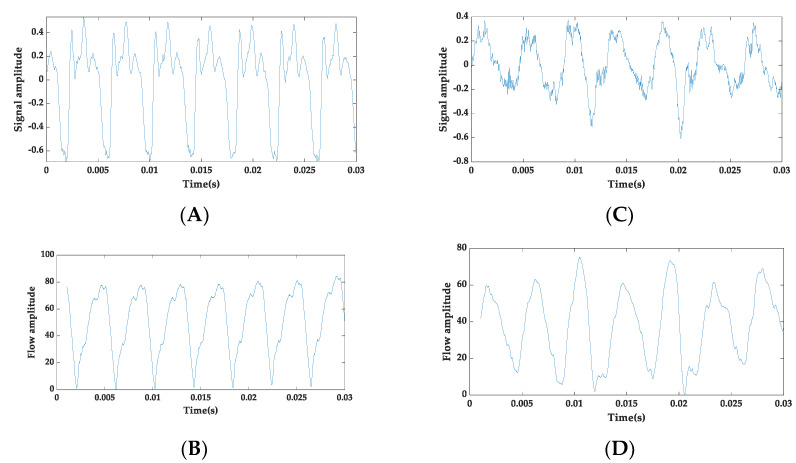
Illustration of speech signal and voice source signal acquired using the IAIF: (**A**) recorded healthy speech signal, (**B**) glottal flow signal of healthy speech, (**C**) recorded pathological speech signal, and (**D**) glottal flow signal of pathological speech.

This paper details a SWT-based framework for assessing voice illnesses. The block diagram in Figure 1 depicts the distinct steps employed in the voice assessment system. The methodology comprises two main phases: (a) learning and (b) evaluation. During the learning stage, a 10-fold cross-validated classification model has been developed using healthful and pathological speech training recordings. In the evaluation phase, unseen speech records are tested on a cross-validated trained model. The normalization of the speech signal amplitude, framing, and windowing are the three main steps in pre-processing. The true voice source is then acquired from the pre-processed voice signal using the GIF method, which isolates the glottal pulse from the spoken signal [35]. In this study, an iterative adaptive inverse filtering (IAIF) technique is applied to obtain the glottal flow and is endorsed due to its robustness in noisy surroundings [36]. In IAIF, the vocal tract response is characterized by the discrete all-pole (DAP) model as it performs better compared to linear predictive coding (LPC) [37]. In an IAIF algorithm, the model of the acoustic tube is estimated in two phases and, hence, is named iterative inverse filtering. In the first step of IAIF, the input speech signal is pre-emphasized. The final refined glottal source is obtained by canceling the influence of lip radiation from the previous stage [38]. Figure 2 gives the illustration of the speech signal and voice source signal acquired using the IAIF. SWT is used to decompose the extracted glottal flow. To represent the voice source in a numerical form, energy and statistical metrics are obtained from each sub-band’s coefficients.

The information gain-centered feature selection method is used to pick the optimal subset of features and omit redundant and irrelevant features. The following section briefly describes the various statistical attributes and SWT. Before feeding the metrics into the classifier, min/max normalization is used to scale all descriptors. This procedure limits the dataset to the range [0, 1]. After normalizing the training data, the min and max values for each attribute of the training data are applied to standardize the test data.

For voice evaluation, two supervised classification algorithms are used: support vector machine (SVM) and stochastic gradient descent (SGD). The typical classifier quality measurement metrics, such as the area under the curve (AUC), classification accuracy (CA), precision (PPV), recall, and F1 score, are calculated. The suggested approach has been validated against four different databases.

Support vector machine (SVM)

An SVM is a linear two-class classifier, and its non-probabilistic facet is a key feature. An SVM partition sample along a decision boundary (plane) is obtained by just a small data subset. The subset of data that supports the decision boundary is adequately termed the support vector. The leftover training samples have no impact on the location of the plane in the feature space. However, the classification model is built in probabilistic classifiers by considering all of the data and thus requiring more computational resources [39].

Furthermore, the binary and linear facets are two constraints of the SVM. The latest developments using “Kernel Trick” handled the linearity constraint of the decision boundary. Additionally, the lack of ability to categorize the data in more than two categories seems to be a topic of current research. The techniques to date support the formation of various SVMs that compare input data to one another in a variety of means, such as one-to-all or all-to-all (the latter is also termed one-to-one) [39,40].

**Figure 3:**
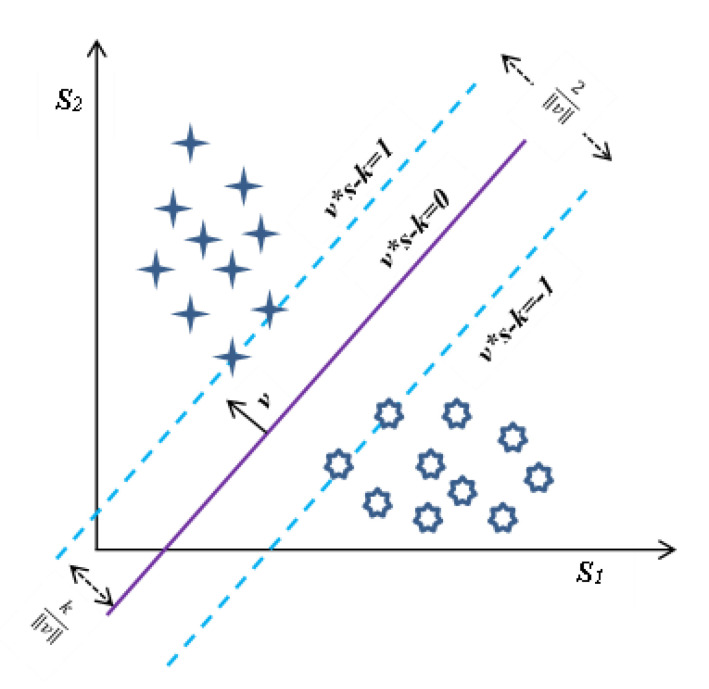
Hyperplanes in SVM for binary classification [39].

We are provided with training data of *n* samples in the form of: $\left({\vec{s}}_{1},t_{1}\right),\ldots \left({\vec{s}}_{n},t_{n}\right)$, where $t_{i}$ is the class label and is either 1 or −1. The label specifies the class of each sample ${\vec{s}}_{i}\;$ to which it belongs and every ${\vec{s}}_{i}$ is a real vector of the x-dimension. While learning, the SVM algorithm determines a decision border inside the input space that splits all data points in the two different classes. In this algorithm, the optimization task is the determination of the linear hyperplane that will have the maximal margin among the two categories. The SVM now employs this hyperplane to evaluate the category of new unseen test samples [40]. The hyperplanes are shown in Figure 3. Thus, the hyperplane splits the set of points ${\vec{s}}_{i}$ for which label $t_{i}=1$ and $t_{i}=-1$. Any hyperplane may be given as a set of data points ${\vec{s}}_{i}$, satisfying the equation:

(1) $$\vec{v}.\vec{s}-k=0,$$

where $\vec{v}$ is the vector perpendicular (normal vector) to the hyperplane and need not be a unit vector. The factor $\frac{k}{\left\|\vec{v}\right\|}$ is used to calculate the offset of a hyperplane from the origin along the normal vector $\vec{v}$. If the input data used to train the SVM are linearly distinguishable, we can select two parallel hyperplanes that split two data classes. Moreover, one has to ensure that the separation of them is as high as possible. The area constrained by two hyperplanes such as these is termed the “margin”, and the max-margin hyperplane is the hyperplane that exists midway between them. These hyperplanes can be characterized by equations with standardized input data [39].

For $\vec{v}.\vec{s}-k=1$, any data points above this plane are of one kind of class with the label 1, and for $\vec{v}.\vec{s}-k=-1$, any data points above this plane are of one kind of class with the label −1. The spacing between these two planes is $\frac{2}{\left\|\vec{v}\right\|}$ and to maximize the margin, we need to minimize the $\left\|\vec{v}\right\|$. We also need to avoid data samples’ falling inside the margin, and this is achieved by adding a constraint: for every *i*, either

$$\vec{v}.{\vec{s}}_{i}-k\geq 1,\;\;\mathrm{if}\;t_{i}=1,\;\mathrm{or}\;\vec{v}.{\vec{s}}_{i}-k\leq 1,\;\;\mathrm{if}\;t_{i}=-1,$$

These restrictions assert that every data value will be fall on the right side of the margin. One can rewrite this as: $t_{i}\left(\vec{v}.{\vec{s}}_{i}-k\right)\geq 1,\;$ for all 1≤i≤n.

To have the optimization problem, it can be put together as: “Minimize $\left\|\vec{v}\right\|$ subject to $t_{i}\left(\vec{v}.{\vec{s}}_{i}-k\right)\geq 1,\;$ for i=1,…n”. The $\vec{v}$ and *k*, which solve this optimization problem and will determine classifier, $\vec{s}\mapsto sgn\left(\vec{v}.\vec{s}-k\right)$. An important consequence of this geometric description is that the max-margin hyperplane is completely determined by those $\vec{s}$ that lie nearest to it. These ${\vec{s}}_{i}\;$are called support vectors [40].

Stochastic gradient descent (SGD)

The SGD is simply an optimization method, but it does not necessarily apply to a particular family of ML models. It is just a means to train a classifier model and is employed in ML for training a wide range of models. However, in this work, it is employed to train the linear SVM, where it plays the role of optimizer [41].

**Figure 4:**
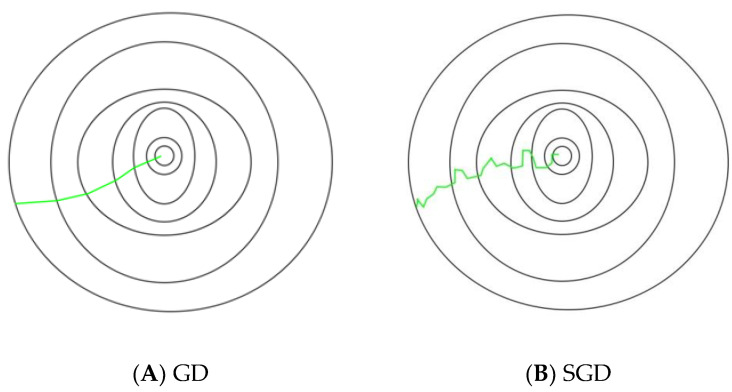
Path followed in GD and SGD to attain minima [43].

The term “batch” refers to the number of samples employed to compute the gradient in each iteration in gradient descents (GD) from a set of data. In classic GD optimization, such as Batch GD, the batch is drawn to be the entire dataset. Although it is very useful to use the entire dataset to obtain the minima in a more noise-free and far less random way, the trouble starts when our dataset is very big. If one is performing a classification task on two million data samples, then all two million samples must be used for one iteration when performing a GD optimization technique, and this will be done for each iteration till the minima are attained. Therefore, this optimization technique is computationally expensive [41]. This issue is resolved by SGD. In SGD, only one sample, i.e., one batch size, is used to execute each iteration. The training sample is randomly mixed and chosen for the iteration. The path followed by this algorithm is generally noisy when reaching the minima compared to classical GD, since in each iteration, a single sample is drawn randomly. However, this noisy path does not affect the performance as long as the algorithm achieves the minima with a considerably smaller training time. The paths followed in GD and SGD are shown in Figure 4. Further, it normally takes relatively high iterations to achieve the minima, due to the random descent. Although it takes a higher number of iterations to achieve the minima than traditional GD, it is still less of a computational burden. Therefore, in most cases, SGD is preferred over traditional GD to optimize the ML algorithm [41]. Let us look at the problem of objective function minimization in the form:

(2) $$P\left(v\right)=\frac{1}{n}\overset{n}{\underset{m=1}{\sum }}P_{m}\left(v\right)$$

where *v* is the parameter that minimizes $P\left(v\right)$.

Every summand function $P_{m}$ corresponds to the *m*th observation of the set of data considered for the training of the model. If the normal GD is employed to minimize the abovementioned function, then it will execute iterations as [42]:

(3) $$v:=v-\eta \nabla P\left(v\right)=v-\frac{\eta }{n}\overset{n}{\underset{m=1}{\sum }}\nabla P_{m}\left(v\right)$$

where the parameter η is the learning rate.

In SGD, the real gradient of $P\left(v\right)$ is approximated by a gradient at the individual observation:

(4) $$v:=v-\eta \nabla P_{m}\left(v\right)$$

When the algorithm runs on through the training data, it conducts the update above for every observation of training. You can make multiple runs over all the training dataset before the algorithm converges. When that is done, the training data (samples) can be shuffled to avoid cycles for each run. Classic frameworks can utilize an adaptive learning rate to converge the algorithm. The SGD may be presented in pseudo code as follows [43]:

- Select an initial vector of parameters v and step size (learning rate) η
- Repeat it until an approximate minimum is obtained:
  ○ Shuffle the observations in the training dataset randomly.
  ○ For m=1,2,3, …,n, do:
    ▪ $v:=v-\eta \nabla P_{m}\left(v\right)$.

#### 2.2.1. Stationary Wavelet Transform

**Figure 5:**
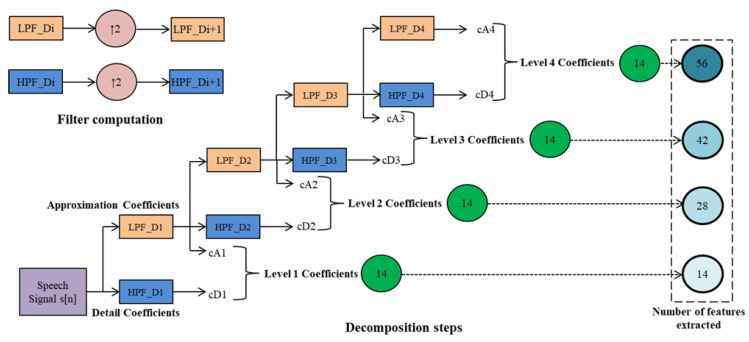
A four-stage decomposition.

In the study of transient phenomena, wavelet transform is widely used to obtain temporal and frequency information from the signal [44,45]. A four-level decomposition of the signal employing the SWT is shown in Figure 5. SWT generates two sets of coefficients for a signal *s[x]* of length *L* in the first stage: approximation coefficients A1 and detail coefficients D1. Convoluting s with the low-pass filter (LF_D) for the approximation component and the high-pass filter (HF_D) for the detail component yields these coefficients. Using a similar strategy, the approximation coefficients A1 are separated into two pieces in the next level. However, in this stage, up-sampling is performed, and *s[x]* is substituted by A1, resulting in A2 and D2. In this research, the glottal wave is decomposed up to level four. The Haar wavelet is used, as the energy and statistical metrics mined from the sub-bands have better discrimination ability compared to higher-order Daubechies wavelets.

#### 2.2.2. SWT Sub-Band Features

As stated earlier, the presented scheme employs wavelet-based energy and statistical attributes extraction. Following SWT decomposition, every sub-band yields a total of seven features. The following is a concise description of the different attributes employed:

1. Energy: the energy of each is calculated using:

(5) $$Energy=\sum_{k=1}^{L}W_{SBi}^{2}$$

The sub-band is denoted by *W_SBi_*, and the number of coefficients is denoted by *L*.

2. Spectral entropy: the sub-band entropy is calculated as follows:

(6) $$Entropy=-\sum_{k=1}^{L}W_{SBi}log(W_{SBi})$$

3. Mean (*μ)*: the mean of both coefficients of the sub-band is calculated as:

(7) $$Mean=\frac{1}{L}\sum_{k=1}^{L}W_{SBi}$$

4. Variance: the variance of both coefficients of the sub-band is calculated as:

(8) $$Variance=\frac{1}{L-1}\sum_{k=1}^{L}{\left|W_{SBi}-\mu \right|}^{2}$$

5. Standard deviation: this is calculated as:

(9) $$Std\_dev=\sqrt{\frac{1}{L-1}\sum_{k=1}^{L}{\left|W_{SBi}-\mu \right|}^{2}}$$

where *μ* denotes the mean of the sub-band.

6. Kurtosis: this is evaluated as:

(10) $$Kurtosis=\frac{E{\left(W_{SBi}-\mu \right)}^{4}}{\sigma^{4}}$$

where *E(i)* denotes the expected value.

7. Skewness: skewness computes the asymmetry of the data in relation to the sample mean and is estimated as:

(11) $$Skewness=\frac{E{\left(W_{SBi}-\mu \right)}^{3}}{\sigma^{3}}$$

#### 2.2.3. Evaluation of Discernment Potential of Attributes

**Figure 6:**
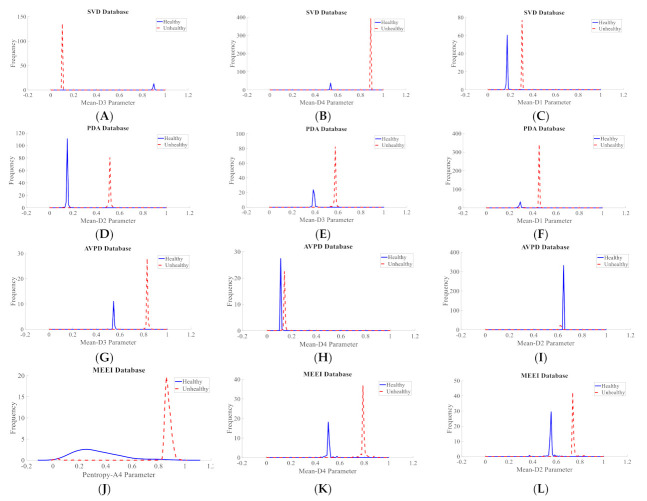
Density plots for the top three descriptors used in the diagnosis of voice illnesses: (**A**) Density plot for Mean-D3 parameter of SVD, (**B**) Density plot for Mean-D4 parameter of SVD, (**C**) Density plot for Mean-D1 parameter of SVD, (**D**) Density plot for Mean-D2 parameter of PdA, (**E**) Density plot for Mean-D3 parameter of PdA, (**F**) Density plot for Mean-D1 parameter of PdA, (**G**) Density plot for Mean-D3 parameter of AVPD, (**H**) Density plot for Mean-D4 parameter of AVPD, (**I**) Density plot for Mean-D2 parameter of AVPD, (**J**) Density plot for Pentropy-A4 parameter of MEEI, (**K**) Density plot for Mean-D4 parameter of MEEI, (**L**) Density plot for Mean-D2 parameter of MEEI.

**Figure 7:**
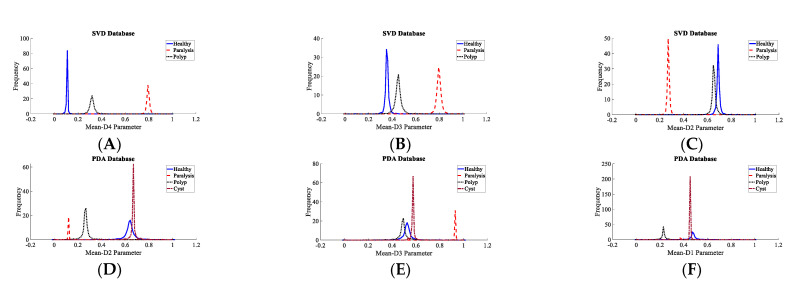

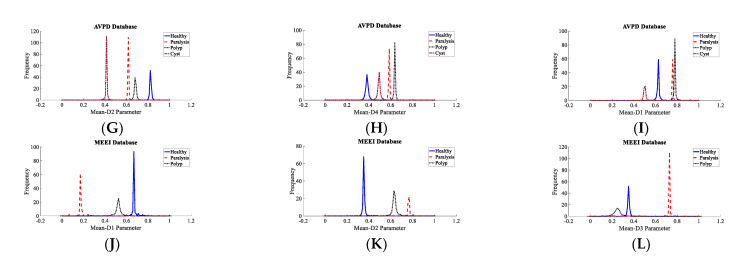
Density plots for top three descriptors for multiclass classification process: (**A**) Density plot for Mean-D4 parameter of SVD, (**B**) Density plot for Mean-D3 parameter of SVD, (**C**) Density plot for Mean-D2 parameter of SVD, (**D**) Density plot for Mean-D2 parameter of PdA, (**E**) Density plot for Mean-D3 parameter of PdA, (**F**) Density plot for Mean-D1 parameter of PdA, (**G**) Density plot for Mean-D2 parameter of AVPD, (**H**) Density plot for Mean-D4 parameter of AVPD, (**I**) Density plot for Mean-D1 parameter of AVPD, (**J**) Density plot for Mean-D1 parameter of MEEI, (**K**) Density plot for Mean-D2 parameter of MEEI, (**L**) Density plot for Mean-D3 parameter of MEEI.

It is extremely important to test the discernment ability of the descriptors before applying to a classifier for a decision to be made. It aids in selecting the best features and removing those that are unwarranted. Subjective and objective methods are used in this study. The probability density plots (PDF) were used to subjectively test the discernment ability of all of the descriptors. The spacing between the peaks in the PDF plots were visually inspected for all four datasets. The greater the distance between the peaks, the greater the discrimination power of the descriptor. For the SVD dataset, it is observed that the spacing between the peaks of the PDF plots of healthy and unhealthy classes are the maximum for the Mean-D3 descriptor, followed by Mean-D4 and Mean-D1. For the PdA dataset, it is observed that the spacing between the peaks of the PDF plots of healthy and unhealthy classes are the maximum for the Mean-D2 descriptor, followed by Mean-D3 and Mean-D1. For the AVPD dataset, it is observed that the spacing between the peaks of the PDF plots of healthy and unhealthy classes are the maximum for the Mean-D3 descriptor, followed by Mean-D4 and Mean-D2. For the MEEI dataset, it is observed that the spacing between the peaks of the PDF plots of healthy and unhealthy classes are the maximum for the Pentropy-A4 descriptor, followed by Mean-D4 and Mean-D2. Figure 6 shows the density plot for these top three descriptors. The density plots of the Mean-D3 feature for SVD dataset is shown in Figure 6A, where Mean-D3 signifies the mean of the detail coefficients of the sub-band after the third decomposition level. One more example, as the PDF plots of the Mean-D2 feature for the PdA dataset, is shown in Figure 6D, where Mean-D2 signifies the mean of the detail coefficients of the sub-band after the second decomposition level. The Mean-D1 and Mean-D4 descriptors represent the mean of the detail coefficients of the sub-band after the first and fourth decomposition levels, respectively. A similar procedure was used to assess the discernment ability of the descriptors for the classification of speech disorders. Figure 7 shows the density plots for the top three features in each dataset for the classification of speech disorders. The density plots for the four classes are plotted for the PdA and AVPD datasets, three of which are pathologic and the fourth is healthful. However, the graphs for three groups are shown in the SVD and MEEI databases, two of which are diseased and the third one is healthful. The quantity of cyst recordings in the SVD and MEEI databases is minimal and of short duration, so only three categories were investigated. Each kind of disorder yields distinct hills with far-flung peaks, implying high discrimination capacity.

The Information Gain (IG) feature-scoring method is used to objectively assess all 56 attributes’ discriminating abilities. Later, the attributes are placed in descending order based on their discernment power. Table 3a,b shows the top 10 attributes with their IG values across each dataset for voice dysfunction identification and classification, respectively. The size of the feature vector depends on the decomposition level. The dimension of the feature vector grows in proportion to the decomposition level. Hence, to reduce the dimension of the feature vector, the feature-selection method is harnessed. It aids in the selection of the best features while eliminating the ones that are redundant. By deleting unnecessary characteristics, feature selection approaches pick a small group of characteristics that boosts the classification rate.

## 3. Results

Two supervised classifiers, SVM and SGD, are used to evaluate vocal abnormalities. The radial basis function (RBF) kernel is utilized in the SVM classifier, and parameter C is set at 1. The hinge as a classification loss function, squared loss as a regression loss function, and ridge (L2) as a regularization approach were chosen as the various parameters for the SGD, also known as linear SVM. A stratiform k-fold cross-validation resampling procedure is more preferable than general cross-validation in terms of bias and variance, so it is used to develop machine learning models for the assessment of voice abnormalities. On four separate datasets, many inter- and intra (cross)-dataset tests were undertaken to determine the utility of the extracted descriptors for voice disease identification and categorization. The acquired glottal wave is dissected up to level 4, and seven descriptors are mined from each sub-band’s approximation as well as detailed coefficients ((7_approximation coefficients + 7_detailed coefficients) × 4 = 56).

### 3.1. Intra-Dataset Voice Pathology Detection Experiments

**Figure 8:**
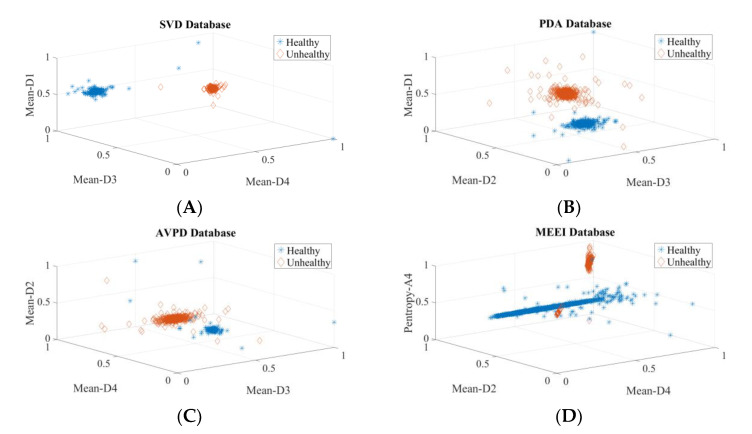
Three-dimensional scatter plots for each database’s top three descriptors: (**A**) Three-dimensional scatter plot of top three descriptors of SVD, (**B**) Three-dimensional scatter plot of top three descriptors of PdA, (**C**) Three-dimensional scatter plot of top three descriptors of AVPD, (**D**) Three-dimensional scatter plot of top three descriptors of MEEI.

Two experiments are carried out to validate the efficacy of extracted wavelet-based features for voice disfunction recognition, one for each of the top three ranked descriptors and one for all 56 descriptors. Table 4 displays the calculated performance-assessing indicators of the classifiers. The observation that the detection rates for the top three and all 56 features are nearly equal leads to the conclusion that only the top three descriptors are vital to detect voice pathology. The suggested system’s computational complexity decreases because of the fewer descriptors and smaller feature vectors. In addition, these results reveal that the features are not noisy, as increasing the number of descriptors has no negative influence on the detection accuracy. The values of classifier performance metrics look identical in many tables because of the very small number of false positives (FP) and false negatives (FN). The scatter plot for these top three descriptors is shown in Figure 8 and clearly shows the power of discrimination as the clusters of healthy and pathological data points (samples) do not overlap. The outliers are not removed; hence, some data points become mixed into other class data points.

### 3.2. Voice Disorder Categorization

Using the information gain (IG) feature ranking method, 12 experiments with distinct sets of selected descriptors are conducted in each database for multiclass classification. These experiments are being carried out to see whether the derived sub-band descriptors can distinguish between healthy people and those with a clinical condition of a vocal cord(s) cyst, paralysis, or polyp. The outcomes of these tests are presented in Table 5, which unmistakably demonstrates that the categorization accuracy (CA) rises as the number of descriptors increases. It assures that the features are not noisy and do not impact the categorization accuracy negatively.

The classification rate with all 56 features and the top five features is nearly identical across all datasets. As a result, it is shown that less than 9% of the entire number of descriptors is sufficient to achieve an almost equal recognition rate. The suggested system’s computational complexity decreases because of the fewer descriptors and smaller feature vectors. The highest accuracy obtained with the AVPD database using the top five descriptors was 99.87%. In the case of the SVD, AVPD, and MEEI databases, the top three descriptors performed extraordinarily. The classification accuracy attained with these top three features is nearly identical to that achieved only with the top five and all 56 features. Figure 3 shows the PDF plots of the top three descriptors, which are not overlapping and endorse the high discrimination capacity.

### 3.3. Dataset-Independent Voice Illness Assessment System

A key objective of this study is to propose a voice pathology identification and classifying framework that is not affected by language, accent, age group, ethnic background, sex, or other factors. There is no single (particular) feature that can provide a high identification and classifying rate across all datasets. Therefore, three common features from the top five features are tactfully picked from all datasets. The chosen three descriptors are Mean-D2, Mean-D3, and Mean-D4. This makes the proposed dataset independent, and as a result, the method is not affected by factors such as sex, age, culture, ethnicity, or language.

**Figure 9:**
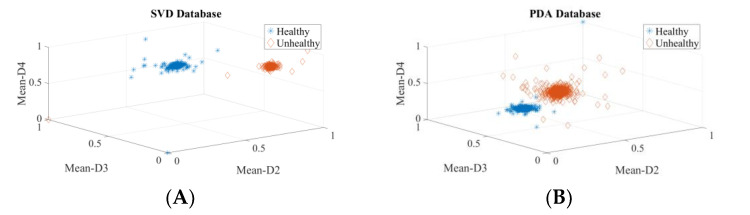

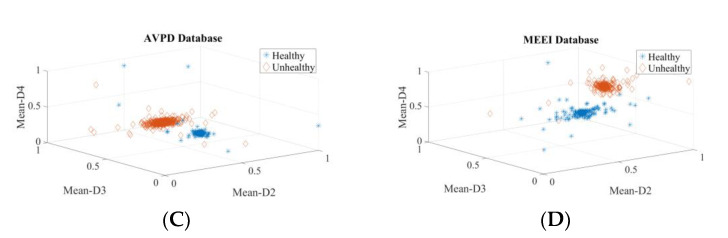
Scatter plots of three most common descriptors for pathology identification process: (**A**) Scatter plot of top three descriptors of SVD, (**B**) Scatter plot of top three descriptors of PDA, (**C**) Scatter plot of top three descriptors of AVPD, (**D**) Scatter plot of top three descriptors of MEEI.

**Figure 10:**
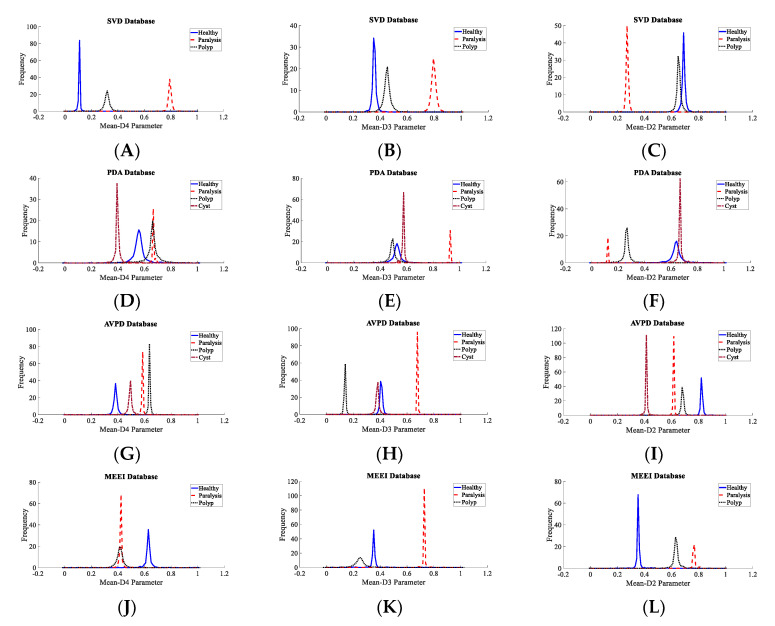
PDF plots of three most common descriptors for multiclass experiments: (**A**) Density plot for Mean-D4 parameter of SVD, (**B**) Density plot for Mean-D3 parameter of SVD, (**C**) Density plot for Mean-D2 parameter of SVD, (**D**) Density plot for Mean-D4 parameter of PdA, (**E**) Density plot for Mean-D3 parameter of PdA, (**F**) Density plot for Mean-D2 parameter of PdA, (**G**) Density plot for Mean-D4 parameter of AVPD, (**H**) Density plot for Mean-D3 parameter of AVPD, (**I**) Density plot for Mean-D2 parameter of AVPD, (**J**) Density plot for Mean-D4 parameter of MEEI, (**K**) Density plot for Mean-D3 parameter of MEEI, (**L**) Density plot for Mean-D2 parameter of MEEI.

To ascertain the discrimination power of these three selected common descriptors, scatter plots and PDF plots are plotted. Figure 9 shows the scatter plots of these descriptors for the SVD, PdA, AVPD, and MEEI datasets for the pathology identification process. In the scatter plot, hardly any of the samples are mixed, indicating that the three descriptors chosen have excellent discrimination ability to separate pathological samples. The quality metrics of the classifiers for speech condition identification with the three most common features are presented in Table 6, and the maximum recognition rate attained is 99.97% for the Arabic dataset. Figure 10 shows the density graphs for the three most common descriptors for speech condition classification. As there are few cyst pathology samples in the SVD and MEEI datasets, three groups—healthy, paralyzed, and polyp—are analyzed. The AVPD and PdA datasets are classified into four categories: normal, cystic, paralyzed, and polyp. The classifiers’ key parameters for categorization are presented in Table 7, and the AVPD dataset attained the best categorization accuracy of 99.87%.

### 3.4. Voice Disorder Detection (Inter-Database)

Inter-database tests are carried out as an extra task to examine the discriminative power of features for voice pathology identification and to ascertain the proposed voice evaluation system’s independence from language, accent, age, social background, sex, and so on. This set of experiments aided in determining how well a voice pathology identification system trained by one dataset would discriminate speech recordings from another dataset. In the inter (cross)-dataset type, 14 experiments are performed using same three most common features, namely, Mean-D2, Mean-D3, and Mean-D4, considered in Section 3.3. The classifier was trained on one of the datasets for the first four tests, and the learned model was tested on the other three datasets individually. For experiments five through ten, all conceivable pairings of two datasets were employed for learning, and the learned model was tested using the leftover two datasets individually. In the last three experiments, every possible combination of the three datasets was used to train the classifier; however, the leftover dataset was used to assess the educated model. The detection rate of these 14 experiments is shown in Table 8 and reveals that the proposed approach is unbiased in terms of spoken language, age, social background, accent, sex, etc.

### 3.5. Voice Pathology Independent System

**Figure 11:**
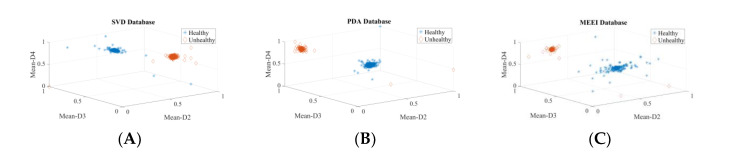
Three-dimensional scatter plots for detection process: (**A**) Three-dimensional scatter plot of SVD for detection process, (**B**) Three-dimensional scatter plot of PdA for detection process, (**C**) Three-dimensional scatter plot of MEEI for detection process.

One more goal of this study is to propose an independent voice pathology system. To ascertain this, extra tests are carried out using variety of pathologies. Up to Section 3.4, all of the experiments are performed using healthy, cyst, paralysis, and polyp speech samples. In the SVD, PdA, and MEEI databases, laryngitis and Reinke’s edema are the most common pathologies, along with paralysis, polyp, and cyst. So, these five pathologies were considered with the healthy speech samples to perform voice identification experiments. The descriptor rankings obtained with these five pathologies are given in Table 9. The descriptor rankings for voice disorder identification shown in Table 3 and Table 9 do not change much. In addition, the descriptors Mean-D1, Mean-D2, Mean-D3, and Mean-D4 have retained their position among the top eight descriptors. The classifier performance measures of this experiment are shown in Table 10. To confirm that the system is independent of voice pathologies, the same three most common descriptors Mean-D2, Mean-D3, and Mean-D4 mentioned in Section 3.3 are used. The highest detection rate obtained is more than 99% in all databases by using only the three most common descriptors. The scatter plots are shown in Figure 11 to justify the high detection rate achieved. It is clear from Figure 11 that these three common descriptors are highly discriminative.

**Figure 12:**
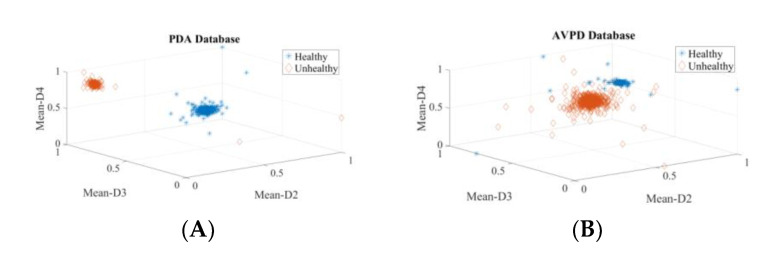
Three-dimensional scatter plots for detection process with full PdA and AVPD datasets: (**A**) Three-dimensional scatter plots for detection process with full PdA dataset, (**B**) Three-dimensional scatter plots for detection process with AVPD dataset.

The PdA databank covers 15 organic and traumatic etiologies and the AVPD database consists of five organic speech pathology samples. All speech samples available in the PdA and AVPD databases are also considered in the next voice disorder identification experiment. This experiment consists of wide variety of voice pathology samples. The top 10 ranked descriptors are shown in Table 11, and it is found that these are almost the same as those listed in Table 3 and Table 9. In addition, the Mean-D1, Mean-D2, Mean-D3, and Mean-D4 descriptors are at rank 1, rank 2, rank 3, and rank 4, respectively. Thus, although the voice pathologies increased compared to earlier experiments, the most discriminatory descriptors remain the same. The classifier performance measures of this experiment are shown in Table 12. Here, two separate experiments are performed with the three most common (Mean-D2, Mean-D3, and Mean-D4) descriptors and all 56 descriptors. Again, the three most common descriptors perform well, and the highest detection rate obtained is 99.99%. The scatter plots for these three common descriptors are shown in Figure 12 and show that the healthy and unhealthy classes’ data points are non-overlapping. The outcome of this experiment ensures that the proposed system is independent of voice pathologies.

Further, two multiclass experiments are performed with PdA and AVPD dataset speech samples. In the PdA dataset multiclass experiment, healthy and seven pathological classes—cyst, paralysis, polyp, laryngitis, Reinke’s edema, nodules, and sulcus—are used. However, in the AVPD dataset multiclass experiment, healthy and five organic pathological classes—cyst, paralysis, polyp, nodules, and sulcus—are used. The top 10 descriptor rankings obtained are shown in Table 13 and are almost identical to the descriptor rankings shown in Table 3. Thus, though the pathologies increased, the ranking of the descriptors does not change much, and the Mean-D1, Mean-D2, Mean-D3, and Mean-D4 descriptors have retained their position in the top 4. The results of these experiments are shown in Table 14 and it is observed that the accuracy obtained with the top 10 and all 56 descriptors is nearly the same as using the SGD classifier. The performance of the system using the three most common descriptors is not as good for the PdA database, as the number of classes in the multiclass experiments is higher compared to the experiments performed in Section 3.2. However, in the AVPD database, the classification rate achieved with the three common (Mean-D2, Mean-D3, and Mean-D4) features is almost identical. Thus, the proposed framework can be used for the assessment of almost any voice pathology that affects the vibratory pattern of vocal folds. Moreover, to perform voice disorder identification or classification, the three most common (Mean-D2, Mean-D3, and Mean-D4) features are adequate to attain sufficient accuracy.

### 3.6. Impact of Wavelet Decomposition Level on the Accuracy of Detecting and Categorization

The feature vector’s dimension is determined by the decomposition level. As the decomposition level grows, so does the dimensionality of the feature vector, increasing the system’s computational complexity and response time. As a result, determining the optimal level of decomposition is required to obtain satisfactory detection and categorization accuracy. Table 15 depicts the impact of the decomposition level on the detection rate and reveals that the detection rate is nearly same even when the decomposition level is increased. Table 16 portrays the impact of the decomposition level on the classifying accuracy, revealing that level 4 achieves the highest classification accuracy. The glottal waveform is decomposed to level 4 in this study because the suggested system achieves both the desired identification and classification accuracy. Table 17 compares the accuracies achieved in this study to those stated in the existing state-of-the-art literature.

## 4. Discussion

This study uses the well-known IAIF method to acquire the glottal wave, which is then dissected using SWT up to level 4. The obtained glottal wave is then numerically quantified using all of the attributes that were mined from each sub-band. The usefulness of extracted features for the identification and categorization of vocal disorders is evaluated using a variety of intra- and inter-database tests. The statistical features have demonstrated outstanding performance for assessing voice diseases. The findings reveal that the descriptors behaved differently for each database, resulting in variations in detection and classification accuracy. The following factors could account for the minor variance in accuracy and other metrics.

1. The voice-acquiring equipment and acoustic settings in each corpus were different.
2. The sampled frequency varies by dataset. This has an impact on the accuracy and reliability of a voice analysis.
3. In the MEEI registry, healthy people were not clinically evaluated. As a result, there is no way of knowing whether these people were truly healthy.
4. Although the recordings are altered to obtain just the stable section of the phonation, the research has revealed that the start and end of the vocalization have more acoustic clues than the steady section.
5. In the German and English datasets, many audios are labeled with multiple diseases.
6. For certain disorders, there are only a few recordings available.
7. The severity of pathology is different in each dataset
8. The distribution of the subject’s age and sex varies by dataset.
9. The SVD dataset does not include relevant information on paralysis, whether unilateral or bilateral.
10. The PdA dataset includes audio files with strong background noise or barely discernible vocalizations.

The wavelet transform has the power to extract spatial–frequency information from a non-stationary signal, and since disordered speech is transient in nature, we used the orthogonal Haar wavelet for multilevel decomposition. We examined Daubechies wavelets over four datasets to validate the use of the Haar wavelet. Table 18 and Table 19 show the calculated detection and classification rates, and Haar outperforms Daubechies wavelets in terms of the classification rate, justifying our choice of Haar wavelets for decomposition. When it comes to detecting voice disorders, both Haar and Daubechies have been proven effective. To confirm that the classifier model developed is independent of the database, it is tested with three more databases other than the database by which it was trained. This set of experiments helped to investigate how efficiently a voice pathology detection system that is educated by one database can distinguish samples from the other database. However, the results were not encouraging. To bring uniformity to the application of the system to various databases, various common descriptors of voice pathologies are identified. The proposed system for identifying and classifying voice disorders is therefore evaluated based on three common features collected from all databases. In terms of the identification and classification accuracy, 99.9% has been attained so far.

## 5. Conclusions

The variation in speech quality due to voice pathology is directly linked to the real biological source of voiced excitations emerging from the glottis. This source of voiced excitations is obtained by IAIF, which gives better insight into the cause of changes in speech quality. For the modeling of the vocal tract, a discrete all-pole model was used in inverse filtering as it performs better compared to LPC in a high-pitched voice. This study proposes a novel voice dysfunction diagnostic and categorization system based on SWT. Multilevel SWT decomposition has been used to detect variation in speech quality, and energy as well as statistical parameters were derived from each sub-band to qualify the glottal wave. The derived parameters are independent of the fundamental frequency and were found to be very successful in distinguishing healthful and disordered speech. The Mean-D1, Mean-D2, Mean-D3, and Mean-D4 descriptors extracted from the detailed coefficients of decomposition level 1, level 2, level 3, and level 4 have very good discrimination power compared to other descriptors for speech dysfunction assessment. The proposed system achieved an average recognition rate of 99.99% and 99.60% for detection and classifying, respectively. The SGD classification model outperformed the SVM classifier, signifying that the extracted features are linearly separable.

The purpose of this study is to create a voice condition assessment system that is independent of voice pathologies as well as the language, accent, age, cultural background, and gender of the speaker by utilizing three common descriptors from the top ten descriptors.

Additionally, this article presents an automated voice disease diagnostic algorithm that is recommended for a wide variety of voice diseases. Our proposed system is among a few speech impairment diagnostic systems that have identified a broad range of laryngeal pathologies. This framework can be introduced as an android app that can be used to check the voice quality in daily life at any instant. The conceived laryngeal pathology evaluation framework can be used to diagnose voice concerns objectively and without invasion. It can be used as a supplemental aid to track the degree of recovery both during and after the cycle of voice therapy.

## Figures and Tables

**Table 1:** Healthy and pathological speakers’ voice (vowel /a/) samples.

Dataset	Normal	Cyst	Paralysis	Polyp	Laryngitis	Reinke’s Edema	Nodules	Sulcus
SVD [32]	262	06	194	44	140	68	--	--
PdA [27]	238	20	18	28	30	29	29	24
AVPD [33]	118	24	45	34	--	--	20	41
MEEI [34]	53	10	52	15	08	53	--	--
In Section 3.1, Section 3.2, Section 3.3 and Section 3.4, speakers’ voice (vowel /a/) samples of healthy, cyst, paralysis, and polyp were used.								
In addition to the speech recording indicated in rows 1 to 4, the following pathological PdA [27] database speech samples were used for experiments in Section 3.5: Acquired iatrogenic trauma on the vocal cords = 2, Upper motor neuron injury = 14, Extrapyramidal alterations = 1, and Lack of closure = 6.								

**Table 2:** Characteristics of the databases.

Sr. No.	Characteristics	SVD [32]	PdA [27]	AVPD [33]	MEEI [34]
1	Language	German	Spanish	Arabic	English
2	Recording place	Institute of Phonetics of the Saarland University, Germany	Príncipe de Asturias Hospital, Madrid	King Abdul Aziz University Hospital, Saudi Arabia	Massachusetts Eye and Ear Infirmary voice and speech laboratory, USA
3	Sampling rate of speech samples	All speech samples 50 KHz	All speech samples 25 KHz	All speech samples 48 KHz	10 KHz, 16 KHz, 25 KHz, and 50 KHz
4	Audio format	.WAV and .NSP	.WAV	.WAV and .NSP	.NSP
5	Recording session(s)	• Sustained vowels /a/, /i/, and /u/ • A sentence	• Sustained vowel /a/	• Vowels /a/, /i/, and /u/ • Al-Fateha (continuous speech) • Arabic digits • Isolated common words	• Sustained vowel /a/ • Rainbow passage (continuous speech)
6	Duration of recorded speech samples	• Vowels: 1–3 s • Sentence: 2 s	Vowels: 1–3 s	• Vowel: 5 s • Al-Fateha: 18 s • Digits: 10 s • Words: 3 s	Healthy • Vowel: 3 s • Passage: 12 s Pathological • Vowel: 1 s • Passage: 9 s
7	Proportion of healthy and pathological subjects	Healthy: 33% Pathological: 67%	Healthy: 54.32% Pathological: 45.68%	Healthy: 51% Pathological: 49%	Healthy: 7% Pathological: 93%
8	Severity of disordered voice quality	Not available	Severity is rated on a GRABS scale	Severity is rated on a scale of 1 (low) to 3 (high)	Not available
9	Voice illness types	Functional and organic	Organic and traumatic etiologies	Organic	Organic and functional
10	Evaluation of normal subjects	No such information is available	No such information is available	Evaluation conducted using laryngeal stroboscope	No such information is available

**Table 3:** (a) Top ten parameters for recognizing voice dysfunction. (b) Top ten parameters for categorizing voice dysfunction.

(a)								
Rank	Detection							
	SVD		PdA		AVPD		MEEI	
	Parameter	IG Value	Parameter	IG Value	Parameter	IG Value	Parameter	IG Value
1	Mean-D4	0.9904	Mean-D3	0.9844	Mean-D3	0.9857	Mean-D4	0.9866
2	Mean-D3	0.9841	Mean-D2	0.9711	Mean-D4	0.9746	Mean-D2	0.9453
3	Mean-D1	0.8880	Mean-D1	0.8929	Mean-D2	0.8959	Pentropy-A4	0.8862
4	Mean-D2	0.5902	Mean-D4	0.6226	Mean-D1	0.8802	Mean-D1	0.8672
5	Pentropy-D2	0.2034	Skewness-D4	0.5664	Kurtosis-A1	0.2414	Mean-D3	0.8611
6	Pentropy-D1	0.2019	Skewness-D3	0.5128	Kurtosis-A2	0.2412	Pentropy-A3	0.7601
7	Pentropy-A2	0.1758	Skewness-D2	0.2123	Kurtosis-A3	0.2391	Pentropy-A1	0.7408
8	Skewness-D4	0.1436	Energy-D1	0.2015	Pentropy-D3	0.2149	Pentropy-A2	0.7178
9	Pentropy-A4	0.1383	Variance-D1	0.2015	Kurtosis-A4	0.2075	Skewness-D2	0.5615
10	Pentropy-D3	0.1282	Std-Dev-D1	0.1736	Pentropy-D4	0.1985	Skewness-D1	0.3802
**(b)**								
**Rank**	**Classification**							
	**SVD**		**PdA**		**AVPD**		**MEEI**	
	**Parameter**	**IG Value**	**Parameter**	**IG Value**	**Parameter**	**IG Value**	**Parameter**	**IG Value**
1	Mean-D4	1.1168	Mean-D1	1.5628	Mean-D2	1.9189	Mean-D1	1.0684
2	Mean-D3	1.0786	Mean-D2	1.5520	Mean-D4	1.8214	Mean-D2	1.0468
3	Mean-D2	0.9874	Mean-D3	1.3169	Mean-D1	1.7703	Mean-D3	0.9598
4	Mean-D1	0.9810	Mean-D4	1.1685	Mean-D3	1.7252	Mean-D4	0.6896
5	Pentropy-A3	0.8053	Variance-A4	0.6850	Mean-A1	0.3323	Pentropy-A4	0.5580
6	Pentropy-A2	0.7660	Variance-A3	0.6830	Mean-A2	0.3323	Pentropy-A3	0.4609
7	Pentropy-A4	0.7356	Variance-A2	0.6821	Mean-A3	0.3323	Kurtosis-D3	0.4108
8	Pentropy-A1	0.6911	Variance-A1	0.6806	Mean-A4	0.3323	Pentropy-A2	0.4023
9	Pentropy-D4	0.5450	Kurtosis-D4	0.6631	Energy-A4	0.2966	Pentropy-A1	0.3881
10	Pentropy-D3	0.4738	Variance-D4	0.6384	Energy-A3	0.2963	Variance-D2	0.3307

**Table 4:** Performance indicators for SVM and SGD classifiers for trials identifying vocal disorders.

Number of Features	Databases	Classifier									
		SVM					SGD				
		AUC	CA (%)	F1 Score	PPV (%)	Recall (%)	AUC	CA (%)	F1 Score	PPV (%)	Recall (%)
All 56	SVD	0.9999	99.55	0.9955	99.55	99.55	0.9930	99.30	0.9930	99.31	99.30
	PdA	0.9999	99.06	0.9906	99.07	99.06	0.9815	98.15	0.9814	98.18	98.15
	AVPD	0.9999	99.87	0.9987	99.87	99.87	0.9997	99.97	0.9997	99.97	99.97
	MEEI	1.0000	99.93	0.9993	99.93	99.93	0.9993	99.93	0.9993	99.93	99.93
Top-3	SVD	0.9999	99.97	0.9997	99.97	99.97	0.9998	99.98	0.9998	99.98	99.98
	PdA	1.0000	99.97	0.9997	99.97	99.97	0.9988	99.88	0.9988	99.88	99.88
	AVPD	0.9999	99.97	0.9997	99.97	99.97	0.9999	99.99	0.9999	99.99	99.99
	MEEI	1.0000	99.93	0.9993	99.93	99.93	0.9999	99.99	0.9999	99.99	99.99

**Table 5:** Performance indicators for SVM and SGD classifiers for multiclass.

Number of Features	Databases	Classifier									
		SVM					SGD				
		AUC	CA (%)	F1 Score	PPV (%)	Recall (%)	AUC	CA (%)	F1 Score	PPV (%)	Recall (%)
All 56	SVD	0.9999	98.62	0.9862	98.68	98.62	0.9904	98.72	0.9872	98.76	98.72
	PdA	0.9998	98.93	0.9893	98.94	98.93	0.9939	99.09	0.9909	99.11	99.09
	AVPD	0.9999	99.85	0.9985	99.85	99.85	0.9983	99.75	0.9975	99.75	99.75
	MEEI	0.9999	99.47	0.9947	99.48	99.47	1.0000	99.99	0.9999	99.99	99.99
Top 5	SVD	0.9999	98.86	0.9886	98.89	98.86	0.9944	99.26	0.9926	99.27	99.26
	PdA	0.9992	98.24	0.9824	98.25	98.24	0.9805	97.08	0.9705	97.11	97.08
	AVPD	0.9999	99.87	0.9987	99.87	99.87	0.9987	99.81	0.9981	99.81	99.81
	MEEI	1.0000	99.65	0.9965	99.65	99.65	0.9986	99.82	0.9982	99.82	99.82
Top 3	SVD	0.9982	98.19	0.9818	98.26	98.19	0.9861	98.15	0.9814	98.18	98.15
	PdA	0.7779	73.83	0.6663	68.49	73.83	0.9628	94.42	0.9439	94.56	94.42
	AVPD	0.9999	99.73	0.9973	99.73	99.73	0.9959	99.39	0.9939	99.40	99.39
	MEEI	1.0000	99.65	0.9965	99.65	99.65	0.9986	99.82	0.9982	99.83	99.82

**Table 6:** SVM and SGD classifier metrics for three most common descriptors.

Databases		Classifier								
		SVM					SGD			
	AUC	CA (%)	F1 Score	PPV (%)	Recall (%)	AUC	CA (%)	F1 Score	PPV (%)	Recall (%)
SVD	0.9999	99.96	0.9996	99.96	99.96	0.9995	99.95	0.9995	99.95	99.95
PdA	0.9999	99.77	0.9977	99.77	99.77	0.9971	99.71	0.9971	99.71	99.71
AVPD	0.9999	99.97	0.9997	99.97	99.97	0.9996	99.96	0.9996	99.96	99.96
MEEI	1.0000	99.86	0.9986	99.86	99.86	0.9993	99.93	0.9993	99.93	99.93

**Table 7:** Performance parameters of classifiers SVM and SGD for three most common descriptors.

Databases	Classifier									
	SVM					SGD				
	AUC	CA (%)	F1 Score	PPV (%)	Recall (%)	AUC	CA (%)	F1 Score	PPV (%)	Recall (%)
SVD	0.9982	98.19	0.9818	98.26	98.19	0.9861	98.15	0.9814	98.18	98.15
PdA	0.9975	97.29	0.9727	97.29	97.29	0.9798	96.97	0.9695	96.99	96.97
AVPD	0.9998	99.87	0.9987	99.87	99.87	0.9989	99.83	0.9983	99.83	99.83
MEEI	0.9999	99.47	0.9947	99.48	99.47	0.9986	99.82	0.9982	99.82	99.82

**Table 8:** Identification rate (%) for cross-dataset experiments with SVM and SGD.

Experiment No.		Classifier							
		SVM				SGD			
	Model Tested on	SVD	PdA	AVPD	MEEI	SVD	PdA	AVPD	MEEI
	Model Trained on								
1	SVD	--	99.98	99.97	99.99	--	99.92	99.98	99.99
2	PdA	99.98	--	99.99	99.99	99.99	--	99.99	99.99
3	AVPD	99.88	99.76	--	99.81	99.99	99.93	--	99.93
4	MEEI	99.86	99.14	99.57	--	99.80	98.89	99.54	--
5	SVD + PdA	--	--	99.99	99.99	--	--	99.99	99.99
6	AVPD + SVD	--	99.99	--	99.99	--	99.99	--	99.99
7	PdA + AVPD	99.99	--	--	99.99	99.99	--	--	99.99
8	MEEI + SVD	--	99.98	99.97	--	--	99.99	99.98	--
9	PdA + MEEI	99.98	--	99.99	--	99.99	--	99.99	--
10	MEEI + AVPD	99.97	99.97	--	--	99.99	99.94	--	--
11	SVD + PDA + MEEI	--	--	99.99	--	--	--	99.98	--
12	SVD + PDA + AVPD	--	--	--	99.99	--	--	--	99.99
13	PDA + MEEI + AVPD	99.99	--	--	--	99.99	--	--	--
14	SVD + MEEI + AVPD	--	98.99	--	--		99.19	--	--

**Table 9:** Top 10 ranked descriptors with healthy and five pathologies.

Rank	SVD	IG Value	PdA	IG Value	MEEI	IG Value
1	Mean-D4	0.9915	Mean-D4	0.9956	Mean-D4	0.9870
2	Mean-D3	0.9901	Mean-D3	0.9906	Mean-D2	0.9788
3	Pentropy-A4	0.9853	Mean-D2	0.9618	Mean-D3	0.9691
4	Pentropy-A1	0.9850	Mean-D1	0.8881	Pentropy-A4	0.9059
5	Pentropy-A2	0.9848	Energy-D3	0.6470	Mean-D1	0.8725
6	Pentropy-A3	0.9847	Variance-D3	0.6470	Pentropy-A3	0.7945
7	Mean-D2	0.9622	Energy-D4	0.6448	Pentropy-A2	0.7448
8	Mean-D1	0.8922	Variance-D4	0.6448	Pentropy-A1	0.7307
9	Pentropy-D4	0.2313	Energy-D2	0.6350	Variance-A4	0.5659
10	Kurtosis-A4	0.1651	Variance-D2	0.6350	Variance-A1	0.5628

**Table 10:** Classifier performance measures for voice pathology identification for SVD, PdA, and MEEI.

Number of Descriptors	Databases	Classifier									
		SVM					SGD				
		AUC	CA (%)	F1 Score	PPV (%)	Recall (%)	AUC	CA (%)	F1 Score	PPV (%)	Recall (%)
All 56	SVD	0.9999	99.94	0.9994	99.94	99.94	0.9995	99.95	0.9995	99.95	99.95
	PdA	0.9999	99.99	0.9999	99.99	99.99	0.9992	99.92	0.9992	99.92	99.92
	MEEI	0.9999	99.99	0.9999	99.99	99.99	0.9988	99.88	0.9988	99.88	99.88
Common 3	SVD	0.9999	99.99	0.9999	99.99	99.99	0.9997	99.97	0.9997	99.97	99.97
	PdA	0.9999	99.99	0.9999	99.99	99.99	0.9999	99.99	0.9999	99.99	99.99
	MEEI	0.9999	99.99	0.9999	99.99	99.99	0.9999	99.99	0.9999	99.99	99.99

**Table 11:** Top 10 ranked descriptors with 15 pathologies from PdA and five from AVPD.

Rank	PdA	IG Value	AVPD	IG Value
1	Mean-D4	0.9962	Mean-D4	0.9956
2	Mean-D3	0.9883	Mean-D2	0.9660
3	Mean-D2	0.9619	Mean-D3	0.9650
4	Mean-D1	0.8768	Mean-D1	0.8829
5	Variance-D3	0.6070	Variance-A4	0.8494
6	Energy-D3	0.6070	Variance-A1	0.8493
7	Variance-D4	0.6012	Variance-A2	0.8493
8	Energy-D4	0.6012	Variance-A3	0.8490
9	Variance-D2	0.5928	Std-Dev-A4	0.8473
10	Energy-D2	0.5928	Std-Dev-A2	0.8470

**Table 12:** Classifier performance measures for voice pathology identification for PdA and AVPD.

Number of Descriptors	Databases	Classifier									
		SVM					SGD				
		AUC	CA (%)	F1 Score	PPV (%)	Recall (%)	AUC	CA (%)	F1 Score	PPV (%)	Recall (%)
All 56	PdA	0.9999	99.99	0.9999	99.99	99.99	0.9994	99.94	0.9994	99.94	99.94
	AVPD	0.9999	99.92	0.9992	99.92	99.92	0.9997	99.97	0.9997	99.97	99.97
Common 3	PdA	0.9999	99.99	0.9999	99.99	99.99	0.9999	99.99	0.9999	99.99	99.99
	AVPD	0.9999	99.99	0.9999	99.99	99.99	0.9997	99.97	0.9997	99.97	99.97

**Table 13:** Top 10 ranked descriptors for multiclass.

Rank	PdA	IG Value	AVPD	IG Value
1	Mean-D2	1.6651	Mean-D2	1.4317
2	Mean-D1	1.3641	Mean-D4	1.4194
3	Mean-D3	1.2962	Mean-D1	1.1262
4	Mean-D4	1.0894	Mean-D3	0.9220
5	Variance-A2	0.7819	Skewness-D4	0.6898
6	Variance-A1	0.7819	Skewness-D3	0.4225
7	Variance-A3	0.7817	Variance-D4	0.3733
8	Variance-A4	0.7784	Energy-D4	0.3714
9	Variance-D4	0.7726	Variance-D3	0.3618
10	Energy-D4	0.7726	Energy-D3	0.3601

**Table 14:** Classifier performance measures of multiclass classification for PdA and AVPD.

Number of Descriptors	Databases	Classifier									
		SVM					SGD				
		AUC	CA (%)	F1 Score	PPV (%)	Recall (%)	AUC	CA (%)	F1 Score	PPV (%)	Recall (%)
All 56	PdA	0.9999	99.01	0.9901	99.01	99.01	0.9899	98.24	0.9825	98.31	98.24
	AVPD	0.9997	97.61	0.9762	97.77	97.61	0.9903	98.38	0.9838	98.45	98.38
Top 10	PdA	0.9135	75.21	0.7063	75.84	75.21	0.9602	93.04	0.9298	93.14	93.04
	AVPD	0.9998	97.58	0.9760	97.77	97.58	0.9868	97.80	0.9779	97.84	97.80
Common 3	PdA	0.9481	73.69	0.6749	74.89	73.69	0.8786	78.76	0.7438	70.88	78.76
	AVPD	0.9991	98.11	0.9811	98.12	98.11	0.9707	95.12	0.9506	95.44	95.12

**Table 15:** Decomposition level effect on identification rate (%).

Database	Classifier									
	SVM					SGD				
	Decomposition Level					Decomposition Level				
	1	2	3	4	5	1	2	3	4	5
SVD	99.89	99.50	99.53	99.55	99.88	99.42	99.24	99.32	99.30	99.35
PdA	99.88	99.65	99.68	99.06	99.91	99.88	98.77	99.80	98.15	99.85
AVPD	99.97	99.96	99.92	99.87	99.96	99.93	99.99	99.98	99.97	99.93
MEEI	99.99	99.99	99.99	99.93	99.99	99.93	99.93	99.93	99.93	99.99

**Table 16:** Decomposition level effect on classifying rate (%).

Database	Classifier									
	SVM					SGD				
	Decomposition Level					Decomposition Level				
	1	2	3	4	5	1	2	3	4	5
SVD	96.75	98.05	98.49	98.62	98.25	96.24	99.19	98.22	98.72	98.69
PdA	94.10	92.14	97.92	98.93	99.04	93.20	96.49	98.93	99.09	99.94
AVPD	95.66	99.55	98.45	99.85	99.85	97.91	99.61	98.62	99.75	99.67
MEEI	97.22	98.95	99.13	99.47	98.95	96.00	99.13	99.65	99.99	98.78

**Table 17:** Comparison with existing approaches.

Ref. No.	Descriptors Used	Conveyed Accuracies (%) for Voice Disorder Identification				
		Database				
		SVD	PdA	AVPD	MEEI	Private
[21]	MFCC	80.20	--	83.65	94.60	--
[29]	IDP	93.20	--	91.50	99.40	--
[30]	Glottal flow descriptors	99.80	99.70	99.80	99.80	--
[46]	Auto-correlation and entropy	92.79	--	99.79	99.69	--
[47]	Peak and lag	90.97	--	91.16	99.80	--
[48]	MDVP	99.68	--	72.53	88.21	--
[49]	Linear prediction of discrete wavelet coefficients	--	--	--	97.01	--
Proposed approach	SWT-based energy and statistical features	99.98	99.98	99.99	99.99	--
		Conveyed accuracies (%) for voice disorder classification				
[28]	Short-time and glottal flow descriptors	--	--	--	--	96.20
[30]	Glottal flow descriptors	90.40	99.30	99.80	90.10	--
[46]	Auto-correlation and entropy features	99.53		96.02	99.54	--
[47]	Peak and lag	98.94	--	95.18	99.25	--
Proposed approach	SWT-based energy and statistical features	99.86	98.93	99.85	99.65	--

**Table 18:** Voice dysfunction detection accuracy (%) using Haar and Daubechies.

Database			SVM							SGD				
	Haar	db2	db4	db6	db8	db10	db12	Haar	db2	db4	db6	db8	db10	db12
SVD	99.55	62.50	73.99	61.80	64.85	61.10	77.05	99.30	99.28	96.91	99.39	98.49	99.12	98.92
PdA	99.06	87.53	82.58	91.00	79.70	72.82	86.62	98.15	98.97	98.06	99.03	93.45	97.55	96.92
AVPD	99.87	95.96	92.57	83.51	85.94	90.94	82.50	99.97	98.90	99.86	99.39	99.00	98.16	99.11
MEEI	99.93	99.72	99.86	99.93	99.79	99.86	99.79	99.93	98.68	99.37	99.79	99.16	99.65	99.37

**Table 19:** Voice dysfunction categorization accuracy (%) using Haar and Daubechies.

Database	SVM							SGD						
	Haar	db2	db4	db6	db8	db10	db12	Haar	db2	db4	db6	db8	db10	db12
SVD	98.62	81.60	87.16	90.01	86.16	91.22	90.75	98.72	95.27	96.51	96.51	95.41	94.37	97.42
PdA	98.93	83.43	86.94	79.72	90.92	81.84	93.25	99.09	95.64	91.98	92.94	91.82	90.92	95.06
AVPD	99.85	71.55	62.16	83.02	59.73	66.34	57.74	99.75	91.06	90.89	93.51	91.95	94.26	87.93
MEEI	99.47	93.75	91.31	94.44	88.71	88.36	86.97	99.99	98.43	95.65	93.57	89.93	92.70	88.71

## Data Availability

The data presented in this study are available on request from the corresponding author.
